# *Haynaldia villosa NAM*-*V1* is linked with the powdery mildew resistance gene *Pm21* and contributes to increasing grain protein content in wheat

**DOI:** 10.1186/s12863-016-0391-4

**Published:** 2016-06-14

**Authors:** Chuanzhi Zhao, Xindi Lv, Yinghui Li, Feng Li, Miaomiao Geng, Yangyang Mi, Zhongfu Ni, Xingjun Wang, Chaojie Xie, Qixin Sun

**Affiliations:** Key Laboratory of Crop Heterosis and Utilization (MOE) and State Key Laboratory for Agrobiotechnology, Key Laboratory of Crop Genomics and Genetic Improvement (MOA), Beijing Key Laboratory of Crop Genetic Improvement, National Plant Gene Research Centre (Beijing), Department of Plant Genetics & Breeding, China Agricultural University, Beijing, 100193 China; Bio-Tech Research Center, Shandong Academy of Agricultural Sciences, Ji’nan, 250100 China

**Keywords:** Wheat, Haynaldia villosa, *NAM* gene, Grain protein content, Pm21

## Abstract

**Background:**

The 6AL/6VS translocation lines, carrying the wheat powdery mildew resistance gene *Pm21*, are planted on more than 3.4 million hectares. The *NAM*-*A1* gene, located on chromosome 6AS of hexaploid wheat, has been implicated with increased wheat grain protein content (GPC). However, the *NAM*-*A1* gene was removed from the 6AL/6VS translocation lines after the original chromosome 6AS was replaced by chromosome 6VS of *Haynaldia villosa*. The present study aimed to clone the *NAM* homologous gene from chromosome 6VS, to analyze the changes of GPC in the 6AL/6VS translocation lines, and to develop related molecular markers for wheat molecular breeding.

**Results:**

A new *NAM* family gene, *NAM*-*V1*, was cloned from 6VS of *H. villosa* (GenBank ACC. no. KR873101). *NAM*-*V1* contained an intact open reading frame (ORF) and putatively encodes a protein of 407 amino acids. Phylogenetic analysis indicated that *NAM*-*V1* was an orthologous gene of *NAM*-*A1*, *B1*, and *D1*. The determination of GPC in four *Pm21* F2 segregation populations demonstrated that the replacement of *NAM*-*A1* by *NAM*-*V1* confers increased GPC in hexaploid wheat. Multiple sequence alignment of *NAM*-*A1*, *B1*, *B2*, *D1*, *D2*, and *V1* showed the single nucleotide polymorphism (SNP) sites for each of the *NAM* genes, allowing us to develop a molecular marker, *CauNAM*-*V1*, for the specific detection of *NAM*-*V1* gene. Our results indicate that *CauNAM*-*V1* can be used as a novel DNA marker for *NAM*-*V1*, and can also be used for selecting *Pm21* in wheat breeding programs. Further, we developed a marker, *CauNAM*-*ABD*, for the amplification and simultaneously distinguish among the *NAM*-*A1*, *NAM*-*B1*, *NAM*-*B2*, *NAM*-*D1*, and *NAM*-*D2* genes in a single step. *CauNAM*-*ABD* enabled us to develop an efficient “one-marker-for-five-genes” procedure for identifying genes and its copy numbers related with grain protein content.

**Conclusion:**

Here, we report the isolation of the *NAM*-*V1* gene of *H. villosa*. This gene contributes to increasing GPC in 6AL/6VS translocation wheat lines. We developed a molecular marker for the specific detection of *NAM*-*V1* and a molecular marker that can be used to simultaneously distinguished among the *NAM*-*A1*, *NAM*-*B1*, *NAM*-*B2*, *NAM*-*D1*, and *NAM*-*D2* genes in a single step.

**Electronic supplementary material:**

The online version of this article (doi:10.1186/s12863-016-0391-4) contains supplementary material, which is available to authorized users.

## Background

Common wheat (*Triticum aestivum*) is one of the most important crop in the word, accounting for about 20 % of the world’s total calorie consumption and providing about 70 million tons of protein every year. Grain protein concentration (GPC) is an important agronomic trait in wheat. Wheat varieties with high gluten and GPC > 12 % are suitable for making bread. Wheats with low gluten content and GPC < 9 % are suitable for making cookies and cakes [1]. It has been established that the GPC of wheat is a quantitative trait that is affected by environmental conditions [2, 3]. Many wild relatives of wheat, including wild emmer wheat (*Triticum turgidum* L. var. *dicoccoides*), have high GPC. In 1991, a complete set of disomic substitution lines were developed by the introgression of each of the chromosomes of wild emmer wheat with high GPC (DIC) into the durum cultivar ‘Langdon’ (LDN). The substitution line in which the chromosome LDN-6B was completely replaced by DIC-6B, showed the highest protein yield [1, 4]. Later, a quantitative trait locus (QTL) for wheat GPC was mapped onto the short arm of chromosome (6BS) using the recombinant inbred lines DIC and LDN [5], and later mapped within a 2.7 cM region as a single Mendelian locus, *Gpc*-*B1* [6]. Uauy et al. (2006) positionally cloned *Gpc*-*B1* and established that it is a member of the NAC transcription factor; they renamed it *NAM*-*B1* [7].

The *NAC* transcript factors are a plant-specific family of transcription factors with a variety of biological functions, including roles in the development of embryos and flowers and responses to biotic and abiotic stress [8–12]. The name *NAC* is related to the *NAM* (No Apical Meristem) gene of Petunia, the *ATAF1* and *ATAF2* (*Arabidopsis* transcription activation factor) of *Arabidopsis*, and the *CUC2* (cup-shaped cotyledon) gene of *Arabidopsis*.

*NAM*-*B1* in wheat is a typical *NAC* transcription factor gene; these genes are highly conserved in maize, rice, barley, and other cereal crops [7]. In addition to the *NAM*-*B1* gene on 6BS, its orthologous genes *NAM*-*A1* on 6AS and *NAM*-*D1* on 6DS, and its homologous genes *NAM*-*B2* on 2BS and *NAM*-*D2* on 2DS have also been identified. The *NAM* genes of wheat are associated with increasing wheat grain protein, zinc, and iron content. The function of *NAM*-*B1*, *B2*, *A1*, *D1*, and *D2* are thought to be largely redundant. The silencing of *NAM* genes resulted in decreases of 30 %, 36 % and 38 % for GPC, iron, and zinc, respectively [7]. Recently, *NAM* orthologous genes have been identified in *Hordeum vulgare* and *Triticum timopheevii* Zhuk; these have been shown to have the same function [1, 13, 14].

*Haynaldia villosa* (2n = 2 × = 14, V genome), belonging to the tribe *Triticeae*, is an annual or perennial diploid plant [15]. As one of the important genetic resources for wheat genetic improvement, *H. villosa* contains many excellent traits, including of resistance to cold, salt, drought, and various wheat diseases, winter hardinesscold, vigorous tillering ability, multi-spikelet morphology, and high grain protein content [16]. Considerable success has been made in transferring beneficial genes from *H. villosa* into wheat via the development and use of substitution and translocation lines. For example, the translocation lines 6AL/6VS carry the powdery mildew resistant gene *Pm21* and showing strong resistance to most of the powdery mildew isolates. The varieties derived from the 6AL/6VS translocation lines are now planted more than 3.4 million hectares [17]. The *Gli*-*V2* gene for k-type sulfur-rich prolamins was also identified from 6VS of *H. villosa* [18, 19]. However, to date, no *NAM* genes have been reported in *H. villosa*. For 6AL/6VS wheat translocation lines, it is unknown about the changes of GPC when the functional *NAM*-*A1* gene on 6A chromosome was removed.

In this study, we report the isolation of the *NAM*-*V1* gene from *H. villosa*. We developed a molecular marker, *CauNAM*-*V1*, which was specific to *NAM*-*V1* and is linked to the powdery mildew resistance gene *Pm21*. In addition, our results showed that *NAM*-*V1* contributes to increasing GPC in hexaploid wheat. We also developed a marker, *CauNAM*-*ABD*, which can amplify and simultaneously distinguish among *NAM*-*A1*, *NAM*-*B1*, *NAM*-*B2*, *NAM*-*D1*, and *NAM*-*D2* in a single step. *CauNAM*-*ABD* enabled the development of an efficient “one-marker-for-five-genes” procedure for identifying genes related with grain protein, zinc, and iron content.

## Results

### Cloning of the *NAM*-*V1* gene

**Fig. 1:**
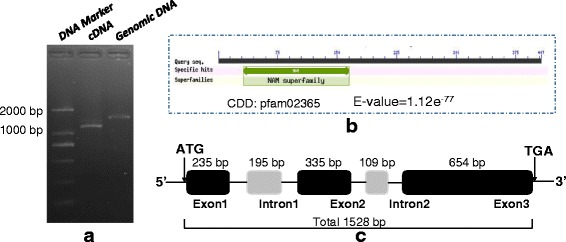
Cloning and sequence analysis of *NAM*-*V1* gene. **a** PCR cloning of *NAM*-*V1* gene. **b** Conserved domain of *NAM*-*V1*. **c** Gene structure of *NAM*-*V1*

The genomic DNA and cDNA of the *NAM*-*V1* gene were amplified from *H. villosa* using two pairs of primers (Fig. 1a). Sequence alignment with known *NAM* genes cataloged in GenBank confirmed that *NAM*-*V1* is a new *NAM* homologous gene (NCBI GenBank ACC. no. KR873101). The *NAM*-*V1* gene had 92 % identity with *NAM*-*A1*, 91 % identity with *NAM*-*D1*, and 90 % identity with *NAM*-*B1. NAM*-*V1* encodes a NAM superfamily domain protein (Fig. 1b). The full-length of *NAM*-*V1* gene is 1,528 bp, and contains three exons and two introns. It is predicted to encodes a protein of 407 amino acids with a molecular weight of 43 KDa (Fig. 1c).

### Phylogenetic analysis of NAM proteins

**Fig. 2:**
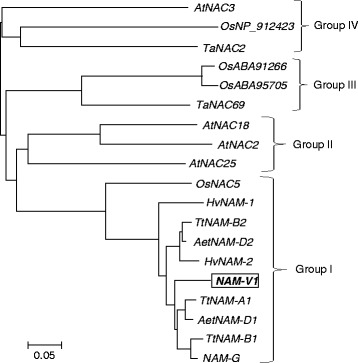
Phylogenic tree of *NAM*-*V1* and other NAC transcription factors from different species. Species abbreviations: *Aegilops tauschii* (Aet), *T. turgidum* (Tt), *Hordeum vulgare* (Hv), *Oryza sativa* (Os), *Arabidopsis thaliana* (At), *Triticum timopheevii* Zhuk (G). GenBank accession numbers: *AtNAC3* (BAB20599), *OsNP*_*912423* (NP_912423), *TaNAC2* (AAU08786), *OsABA91266* (ABA91266), *OsABA95705* (ABA95705), *TaNAC69* (AAY44098), *AtNAC18* (NP_175696), *AtNAC2* (NP_188170), *AtNAC25* (NP_564771), *OsNAC5* (NP_911241), *HvNAM*-*1* (DQ869678), *TtNAM*-*B2* (DQ869676), *AetNAM*-*D2* (DQ869677), *HvNAM*-*2* (DQ869679), *TtNAM*-*A1* (DQ869672), *AetNAM*-*D1* (DQ869675), *TtNAM*-*B1* (DQ869673), *NAM*-*G* (AEI98797)

A neighbor-joining phylogenetic tree was deduced using MEGA 6.0 based the predicted amino acid sequences of NAM-V1 and NAM family proteins of other species (Fig. 2). A total of nineteen proteins were classified into four groups. NAM-V1 belongs to group I, the largest group (10 genes). Group I also includes NAM-A1 encoded by a gene on chromosome 6A and NAM-B2 from chromosome 2B of durum wheat (*T. turgidum* var. *durum*), NAM-B1 from chromosome 6B of wild emmer wheat (*Triticum. turgidum* L. var. *dicoccoides*), NAM-D1 from chromosome 6D and NAM-D2 from chromosome 2D of *Aegilops tauschii*, HV-NAM1 and HV-NAM2 from the H genome of *Hordeum vulgare*, and NAM-G from the G genome of *Triticum timopheevii* Zhuk. The phylogenetic tree showed that *NAM*-*V1* belonged to the same group with *NAM*-*A1*, *B1* and *D1*, the orthologous genes from the sixth chromosomes of the A, B, and D sub genomes, respectively. NAM-B2 and NAM-D2, encoded by genes on the second chromosomes of the B and D sub genomes, respectively, were also close to *NAM*-*V1*. It has been shown that *NAM*-*A1*, *B1*, *B2*, *D1*, and *D2* all function in the regulation of grain protein content, iron, and zinc. Therefore, it is reasonable to speculate that the intact *NAM*-*V1* gene might encode a protein with a similar function. There were three proteins in group II; all three are from *Arabidopsis*. Among these AtNAC2 is associated with lateral root development [20]. Group III included three proteins. In this group, OsABA91266 and OsABA95705 come from *Oryza sativa. TaNAC69* come from wheat, which responses to cold, drought and salt stress, and being associated with the adaptability of wheat under stress conditions [21]. Group IV also included three genes, that was *TaNAC2* of wheat, *OsN*_*NP 912423* of *Oryza sativa* and *AtNAC3* of *Arabidopsis*. Both *TaNAC2* and *OsN*_*NP 912423* were related to stress tolerance [22, 23].

### Sequence alignment and molecular maker development

**Fig. 3:**
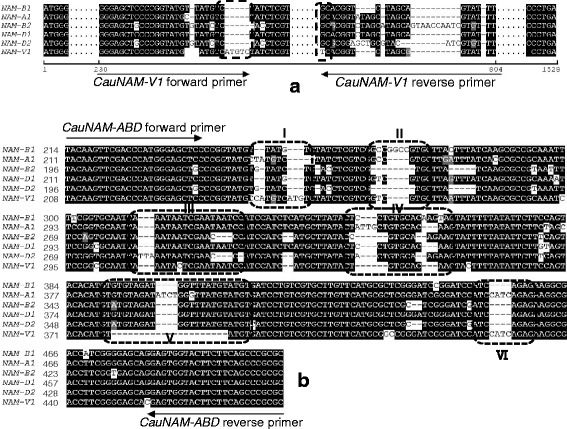
DNA alignment and design of specific molecular markers. **a** Specific molecular marker for the detection of *CauNAM*-*V1*. **b** Polymorphic sites and specific molecular marker *CauNAM*-*ABD* for distinguishing among *NAM*-*A1*, *NAM*-*B1*, *NAM*-*B2*, *NAM*-*D1*, *NAM*-*D2*, and *NAM*-*V1*

In order to develop specific markers for the detection of the *NAM*-*V1* gene and other *NAM* genes in hexaploid common wheat, a multiple sequence alignment was conducted (Fig. 3, Additional file 1: Figure S1). Multiple sequence alignment of *NAM*-*A1*, *B1*, *B2*, *D1*, *D2*, and *V1* showed that there was a specific “ATGTC” insert at the 247th nucleotide of *NAM*-*V1*. The “G to T” single nucleotide polymorphism (SNP) was only observed in the *NAM*-*V1* gene at 785th nucleotide (Fig. 3a). These polymorphic sites were introduced into the 3′ region of the forward and reverse primers, allowing us to develop a specific molecular marker, “*CauNAM*-*V1*”, for the *NAM*-*V1* gene.

We also found a region rich in polymorphism from the 240th -430th nucleotide of the *NAM* genes; this region contained six obvious polymorphic sites (Fig. 3b). A pair of primers, “*CauNAM*-*ABD*”, for the simultaneous detection and discrimination among all of these *NAM* genes, was designed according to the sequence alignment results. For *NAM*-*A1*, *B1*, *B2*, *D1*, *D2*, and *V1*, the expected lengths of the amplification products were 294 bp, 290 bp, 265 bp, 283 bp, 270 bp and 270 bp, respectively.

### Molecular marker *CauNAM*-*V1* is specific for the *NAM*-*V1* gene and is linked with powdery mildew resistance gene *Pm21*

**Fig. 4:**
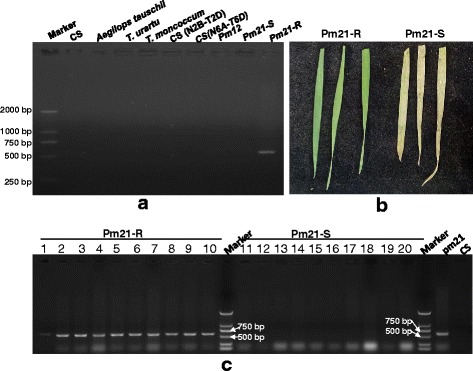
Specific molecular marker for the *NAM*-*V1* gene. **a** PCR amplification of the *NAM*-*V1* gene using a specific molecular marker in different wheat materials. **b** Identification of the resistance of *Pm21* segregation population to powdery mildew, **c** PCR detection using the *CauNAM*-*V1* specific molecular marker in the segregation population resistant to powdery mildew

To test if the molecular marker *CauNAM*-*V1* was specific for the *NAM*-*V1* gene, it was used with DNA from common wheat Chinese Spring (CS), *Aegilops tauschii*, *T. urartu*, *T. mononcoccum*, Chinese Spring nullisomic-tetrasomic lines CS N2B-T2D and CS N6A-T6B, susceptible and resistance individuals from F2 a segregation population of *Pm21*, and a wheat cultivar carrying *Pm12. CauNAM*-*V1* was able to amplify a product only in the materials carrying *Pm21* that contained the 6VS chromosome of *H. villosa* (Fig. 4a). According to the powdery mildew resistance identification results (Fig. 4b), ten resistant individuals and ten susceptible individuals were used for amplification via *CauNAM*-*V1*. Using *CauNAM*-*V1*, a product was amplified from all of the tested resistant individuals; no product was amplified from any of the tested susceptible individuals (Fig. 4c). Thus the marker *CauNAM*-*V1* is linked to *Pm21*. These experiments also indicate that *NAM*-*V1* comes from chromosome 6 V, not from 6A or 6D.

### Detection of *NAM*-*A1*, *B1*, *D1*, *D2*, and *B2* using *CauNAM*-*ABD*

**Fig. 5:**
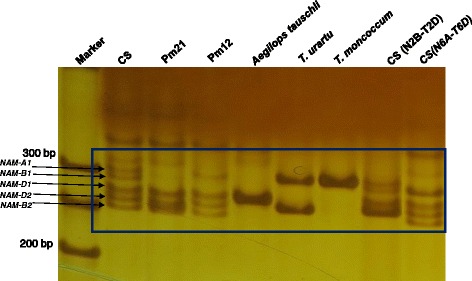
The amplification of primer *CauNAM*-*ABD* in different species. Line 1 to line 9 represented Chinese Spring (CS), 6AL/6VS translocation of wheat carrying powdery mildew resistant gene *Pm21*. 6BL/6SS translocation of wheat carrying powdery mildew resistant gene *Pm12*, *Aegilops tauschii*, *T. urartu*, *T. moncoccum*, CS (N2B-T2D) and CS (N6A-T6D), respectively

Using *CauNAM*-*ABD*, five specific products with different sizes were amplified from common wheat Chinese Spring (CS); these products represented *NAM*-*A1* (294 bp), *B1* (290 bp), *D1* (283 bp), *D2* (270 bp) and *B2* (265 bp), respectively (Fig. 5). For *Pm21* and *Pm12*, there were no bands for *NAM*-*A1* or *NAM*-*B1*, owing to the deletion of 6AS and 6BS, respectively. Only one specific amplification band could be detected with *T. moncoccum* (A^m^ genome). In the Chinese Spring nullisomic-tetrasomic lines CS N6A-T6B, the band representing the *NAM*-*A1* product were not be observed. The band for the *NAM*-*B1* product was brighter than the other bands because there are two copies of the *NAM*-*B1* gene in CS N6A-T6B. The same phenomenon was also observed in CS N2B-T2D, suggesting the *CauNAM*-*ABD* can also measure the copy number of *NAM* genes. Thus, *CauNAM*-*ABD* can amplify and distinguish *NAM*-*A1*, *NAM*-*B1*, *NAM*-*B2*, *NAM*-*D1*, and *NAM*-*D2* in hexaploid common wheat.

### Correlation analysis of GPC and genotype

**Fig. 6:**
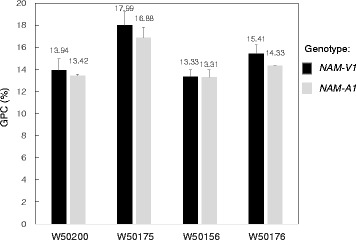
Measurement of grain protein concentration (GPC) of four *Pm21* segregation populations. Four *Pm21* segregation populations (W50200, W50175, W50156, and W50176) were constructed. Genotypes were determined using the molecular marker *CauNAM*-*V1*. The GPC was measured by NIRS. Three replications were analyzed for each accession

In order to analyze the contribution of *NAM*-*V1* and *NAM*-*A1* to GPC, four *Pm21* F2 segregation populations (W50200, W50175, W50156, and W50176) were constructed. The average GPC for the *NAM*-*V1*/*NAM*-*A1* genotypes in W50200, W50175, W50156, and W50176 were 13.94 %/13.42 %, 17.99 %/16.88 %, 13.33 %/13.31 % and 15.41 %/14.33 %, respectively (Fig. *6*). The GPC of the individuals containing the *NAM*-*V1* gene were higher than those containing the *NAM*-*A1* gene in all four of the segregation populations. The average increasing of GPC were 0.52 %, 1.11 %, 0.02 % and 1.08 % in four populations. These results suggest that *NAM*-*V1* contributes to increasing GPC in 6AL/6VS translocation lines of hexaploid wheat.

## Discussion

In developing countries, malnutrition caused by the lack of one or many kinds of trace elements is affecting more than 20 million people. In some areas, as many as 47 % of preschool children suffer from iron deficiency, resulting in poor physical and mental development. Malnutrition caused by zinc deficiency affects about 10 million people worldwide. Zinc deficiency can also cause retarded growth and can destroy the body’s immune system [24]. The *NAM*-*B1* gene from wild emmer wheat and the *NAM*-*A1*, *NAM*-*D1*, *NAM*-*B2*, and *NAM*-*D2* genes from durum wheat not only affect the protein content of wheat grain, the expression levels of these genes are also positively correlated with the iron and zinc levels in grain [7]. In this study, a new homologous gene of *NAM*-*B1*, *NAM*-*V1*, was cloned from *H. villosa*. Gene structure analysis showed that the gene had a complete open reading frame, suggesting that *NAM*-*V1* is a functional gene.

Molecular marker-assisted selection plays an important role in current crop breeding methods, especially in plant disease-resistance breeding. To date, about 70 powdery mildew resistance genes have been identified. One of these is *Pm21*, an effective disease resistance gene for most of the physiological races of the fungal pathogen *Blumeria graminis* f. sp. *Tritici* [17]. *Pm21* and *NAM*-*V1* were all identified from 6VS of *H. villosa*. Because chromosome synapsis did not occur between 6AS of *H. villosa* and 6AS of common wheat during meiosis [25], the specific marker *CauNAM*-*V1* can be used to detect *Pm21* and loci on 6VS that may be associated with other agronomic traits such as the k-type sulfur-rich prolamins gene *Gli*-*V2*. Previous studies have shown that *NAM*-*A1*, *NAM*-*B1*, *NAM*-*B2*, *NAM*-*D1*, and *NAM*-*D2* are all functional genes that are highly conserved in hexaploid common wheat [7]. Additionally, owing to sequence similarity, it has been difficult to discriminate the genotype of *NAM* genes. Here, we developed a molecular marker that can simultaneously amplify *NAM*-*A1*, *NAM*-*B1*, *NAM*-*B2*, *NAM*-*D1*, and *NAM*-*D2*. The genotype and gene copy numbers can be estimated according the electrophoresis results, providing a useful method for screening high grain protein, zinc, and iron content wheat varieties.

*Pm21* is one of the most effective resistance genes against powdery mildew. The 6AL/6VS translocation lines of hexaploid wheat, which carry *Pm21*, has been widely applied in wheat breeding programs. However, it is unclear whether the changes in GPC that occur following the introgression of chromosome segments of *H. villosa*, when original function gene *NAM*-*A1* in 6A chromosome was removed. Here, we isolated the *NAM*-*V1* gene from *H. villosa* and showed that *NAM*-*V1* is an intact and likely functional gene in 6AL/6VS translocation lines of hexaploid wheat. In common wheat, *NAM*-*A1* is known to be a functional gene. Our results demonstrate that the replacement of *NAM*-*A1* by *NAM*-*V1* confers increased grain protein content, implying that *NAM*-*V1* is more efficient than *NAM*-*A1* in increasing the GPC. The differences in the efficiency between *NAM*-*V1* and *NAM*-*A1* might be affected by many factors such as gene structure, gene expression levels, and/or promoter sequences. In addition, this study showed that the powdery mildew resistant genes *Pm21* and *NAM*-*V1* are responsible for co-segregating traits in wheat 6AL/6VS translocation lines. Therefore, the specific marker *CauNAM*-*V1* can also be used for selecting both disease resistance and high GPC genotypes in wheat breeding programs. *CauNAM*-*V1* is a dominant molecular marker that can be easily detected by agarose gel electrophoresis. *CauNAM*-*V1* should help efforts to utilize disease resistance and high protein genes from 6VS of *H. villosa* in wheat improvement programs.

## Conclusions

Here, we report the isolation of the *NAM*-*V1* gene of *H. villosa*. This gene contributes to increasing GPC in 6AL/6VS translocation wheat lines. We developed a molecular marker for the specific detection of *NAM*-*V1* and a molecular marker that can be used to simultaneously distinguished among the *NAM*-*A1*, *NAM*-*B1*, *NAM*-*B2*, *NAM*-*D1*, and *NAM*-*D2* genes in a single step.

## Methods

### Plant material and fungal isolates

The einkorn wheat cultivars *T. urartu* (A^u^) and *T. mononcoccum* (A^m^) were obtained from the Plant Germplasm Institute of Kyoto University (Japan). Powdery mildew isolate E09 was provided by Prof. Xiayu Duan of the Institute of Plant Protection of the Chinese Academy of Agricultural Sciences. The wheat lines 2 N1862 (containing the powdery mildew resistance gene *Pm12*) and W50200 (containing the powdery mildew resistance gene *Pm21*), as well as the common wheat cultivars Chinese Spring and Xuezao are kept at our laboratory. Two Chinese Spring nullisome-tetrasomic lines for homeologous group 2 (CS N2B-T2B) and homeologous group 6 (CS N6A-T6B) were kindly provided by Drs. W. J. Raupp and B. S. Gill of the Wheat Genetics Resource Centre of Kansas State University, USA.

### Powdery mildew resistance identification

Powdery mildew resistance identification was performed as described in a previous study [26]. The reaction of seedlings to powdery mildew were scored on 0 (no visible symptoms), 0; (necrotic flecks), 1 (necrosis with low sporulation), 2 (necrosis with medium sporulation), 3 (no necrosis with medium to high sporulation) and 4 (highly susceptible reactions).

### GPC determination

According to a method described in previous studies [27, 28], the grain protein content (GPC) in mature seeds from the *Pm21* segregation population was measured by near-infrared reflectance spectroscopy (NIRS) on a Perten DA 7200 instrument (Perten Instruments, Sweden). Three replicates were analyzed for each accession.

### Preparation of template DNA

Genomic DNA was extracted from leaves using a cetyl trimethylammonium bromide (CTAB) method [29]. Total RNA was isolated using RNAiso plus Reagent (Takara, Japan) according the manufacturer’s instructions and purified using DNase I. Complementary DNA was synthesized using a PrimeScript 1st Strand cDNA kit (Takara, Japan).

### Primer design and PCR Amplification

The DNA sequences of *NAM*-*A1* (DQ869672), *NAM*-*D1* (DQ869675), *NAM*-*B1* (DQ869673), *NAM*-*B2* (DQ869676), and *NAM*-*D2* (DQ869677) were obtained from GenBank (http://www.ncbi.nlm.nih.gov/genbank). Primer pairs were designed according to the DNA sequences of *NAM* genes for genomic DNA cloning (*NAMORF1*) and cDNA cloning (*NAMORF2*). The forward and reverse primers contained the initiation codon and termination codon, respectively (Table 1). PCR reactions were performed as follows: 94 °C for 3 min, followed by 35 cycles at 94 °C for 30 s, 58-60 °C for 30 s, and 72 °C for 2 min, with a final extension at 72 °C for 7 min. The PCR products were separated on 1.0 % agarose gels and photographed under UV light. After electrophoresis, the specific band was excised from the gel and ligated into pMD18-T vector for transformation. For the detection of molecular marker *NAM*-*ABD*, the PCR products were separated on 8 % non-denaturing polyacrylamide gels. Gels were fast silver stained and photographed. The positive clones for sequence determination were screened by PCR using M13 primers.

**Table 1:** Primers used in this study

Primer name	Primer sequence (5′-3′)	Product length	Annealing Temperature	Purpose
*NAMORF1*	F: GATGAGGTCCATGGGCAG	1528 bp	60 °C	Genomic DNA cloning
	R: TCATTTGCTCAGGGATTCC			
*NAMORF2*	F: ATGGGCAGCTCCGACTCA	1540 bp	60 °C	cDNA cloning
	R: TCAGGGATTCCAGTTCACG			
*CauNAM*-*V1*	F: TCCCCGGTATGCCATGTC	575 bp	58 °C	Specific molecular marker for *NAM*-*V1*
	R: AAGATACCGCTAGACCGTGA			
*CauNAM*-*ABD*	F: TACAAGTTCGACCCATGGGA	265 -294 bp	58 °C	Molecular marker for *NAM*-*A1*, *B1*, *D1*, *B2* and *D*2
	R: GCGCGGGCTGAAGAAGTA			

### Bioinformatics analysis of the *NAM*-*V1* gene

The open reading frame (ORF) of the *NAM*-*V1* gene was predicted using the NCBI online ORF Finder (http://www.ncbi.nlm.nih.gov/gorf/gorf.html). The molecular weight and isoelectric point were predicted using DNAstar software (http://www.dnastar.com). The conserved domain (s) were predicted by alignment with NCBI CDD database (http://www.ncbi.nlm.nih.gov/cdd). Multiple sequence alignments were analyzed using ClustalW software (http://www.ch.embnet.org/software/ClustalW.html). Multiple-alignment files were shaded using BOXSHADE 3.2 (http://www.ch.embnet.org/software/BOX_form.html). Phylogenetic analysis conducted with MEGA 6 (www.megasoftware.net/).

## Abbreviations

6AS, short arm of chromosome 6A; 6BS, short arm of chromosome 6B; CS, common wheat cultivar Chinese Spring; GPC, grain protein content; NAM, no apical meristem; QTL, quantitative trait locus; SNP, single nucleotide polymorphism

## Additional file

Additional file 1: Figure S1. Multiple alignments of deduced amino acid sequences of the NAM proteins. (RTF 117 kb)
