# Supplementary material for: Haynaldia villosa NAM-V1 is linked with the powdery mildew resistance gene Pm21 and contributes to increasing grain protein content in wheat
Source: BMC Genet. 2016 Jun 14;17:82. doi: 10.1186/s12863-016-0391-4 (PMC4908707; doi:10.1186/s12863-016-0391-4)
Supplement: Additional file 1: Figure S1. — Multiple alignments of deduced amino acid sequences of the NAM proteins. (RTF 117 kb) [file 12863_2016_391_MOESM1_ESM.rtf]

NAM-A1    1 MGSSDSSSGSAQKAARHQHEP--PPPRQRGSAPELPPGFRFHPTDEELVVHYLKKKAAKVPLPVTIIAEVDLYKFDPWELPEKATFGEQE
NAM-B1    1 MGSSDSSSGSAQKATRYHHQHQ-PPPPQRGSAPELPPGFRFHPTDEELVVHYLKKKADKAPLPVNIIAEVDLYKFDPWELPEKATIGEQE
NAM-H1    1 MGSPDSSSGSAQKPPRHQHQHQPPPPRRQGSAPELPPGFRFHPTDEELVVHYLKKKAAKAPLPVTIIAEVDLYKFDPWELPEKATFGEHE
NAM-B2    1 MGSSDSSSGSAPPRHQ-------PPPPQQGSAPELPPGFRFHPTDEELVVHYLKKKAAKVPLPVTIITEVDLYKFDPWELPEKATFGEQE
NAM-D1    1 MGSSDSSSGSAQKAARHQHEP--PPPRQRGSAPELPPGFRFHPTDEELVVHYLKKKAAKVPLPVTIIAEVDLYKFDPWELPEKATFGEQE
NAM-D2    1 MGSSDSSSGSAPPRHQ-------PPPPQQGSAPELPPGFRFHPTDEELVVHYLKKKAAKVPLPVTIIAEVDLYKFDPWELPEKATFGEQE
NAM-H2    1 MGSSDSSSGSAPPRHQ-------PPPPQQGSAPELPPGFRFHPTDEELVVHYLKKKAAKVPLPVTIIAEVDLYKFDPWELPEKATFGEQE
NAM-V1    1 MGSSDSSSGSARKATRHQHQP--PPS-QRGSAPELPPGFRFHPTDEELVVHYLKKKAAEVPLPATIIAEVDLYKFDPWELPEKATFGEHE

NAM-A1   89 WYFFSPRDRKYPNGARPNRAATSGYWKATGTDKPILASGTGCGLVREKLGVKKALVFYRGKPPKGLKTNWIMHEYRLTDVSGSTTTSRPP
NAM-B1   90 WYFFSPRDRKYPNGARPNRAATSGYWKATGTDKPILASGTGCGLVREKLGVKKALVFYRGKPPKGLKTNWIMHEYRLTDASGSTTATNRP
NAM-H1   91 WYFFSPRDRKYANGARPNRAATSGYWKATGTDKPILASATGCG--REKVGVKKALVFYRGKPPRGLKTNWIMHEYRLTGASAGSTTTSRP
NAM-B2   84 WYFFSPRDRKYPNGARPNRAATSGYWKATGTDKPILAS----GCGREKVGVKKALVFYRGKPPKGLKTNWIMHEYRLTDASSSATTSRPP
NAM-D1   89 WYFFSPRDRKYPNGARPNRAATSGYWKATGTDKPILASGTGCGLVREKLGVKKALVFYRGKPPKGLKTNWIMHEYRLTDASGSTTTSRPP
NAM-D2   84 WYFFSPRDRKYPNGARPNRAATSGYWKATGTDKPIMAS----GCGREKVGVKKALVFYRGKPPKGLKTNWIMHEYRLTDASSSATTSRPP
NAM-H2   84 WYFFSPRDRKYPNGARPNRAATSGYWKATGTDKPILAS----GCGREKVGVKXALVFYRGKPPKGLKTNWIMHEYRLTDASSSAATSRPP
NAM-V1   88 WYFFSPRDRKYPNGARPNRAATSGYWKATGTDKPILASGPVCGPGREKLGVKKALVFYRGKPPKGLKTNWIMHEYRLTDASGSTTPCRPP

NAM-A1  179 PP--VTGGSRAAASLRLDDWVLCRIYKKINKAAAGD--QQRSTECEDSVEDAVTAYPLYATAGMAGAGAHGSNYASPSLLHH-QDS-HFL
NAM-B1  180 PP--VTGGSRAAASLRLDDWVLCRIYKKINKAAAGD--QQRNTECEDSVEDAVTAYPLYATAGMTGAGAHGSNYASPSLLHH-QDS-HFL
NAM-H1  179 PP--VTGGSRAPASLRLDDWVLCRIYKKTSKAAAAVGDEQRSMECEDSVEDAVTAYPPYATAGMAGAGAHGSNYVQLLHHHDSHEDNFQL
NAM-B2  170 PVT----GGSRSASLRLDDWVLCRIYKKINKAAAGD--QQRSMECEDSVEDAVTAYPLYATAGMTGAGAHGSNYDSLLHHQDSHED-NFL
NAM-D1  179 PPPPVTGGSRAAASLRLDDWVLCRIYKKINKAAAGD--QQRSMECEDSVEDAVTAYPLYATAGMAGAGAHGSNYASSSLLHH-QDS-HFL
NAM-D2  170 PVT----GGSRAASLRLDDWVLCRIYKKINKAAAGD--QQRSMECEDSVEDAVTAYPPYATAGMTGAGAHGSNYDSLLHHQDSHED-NFL
NAM-H2  170 PVT----GGSRAASLRLDDWVLCRIYKKINKAAAAD--QQRSMECEDSVEDAVTAYPPYATACMTGEGAHGSNYASLLHHQDSHED-NFL
NAM-V1  178 P---VIGGSRAAASLRLDDWVLCRIYKKINKAAAGD--QQRSTECEDSVEDAVTAYPLYATAGMAGAGAHGSIYASPSLLHH-QDS-HFL

NAM-A1  263 EGLFTADDAGLSAGATSLSHLAAAARASPAPTKQFLAPSSSTPFNWLDASPAG-ILPQARNFPGFNRSRNVGNMSLSSTADMAG----AA
NAM-B1  264 DGLFTADDAGLSAGATSLSHLAAAARASPAPTKQFLAPSSSTPFNWLDASPVG-ILPQARNFPGFNRSRNVGNMSLSSTADMAG----AV
NAM-H1  267 DGLLTEHDVGLSAGAASLGHLAAAAR----ATKQFLAPSSSTPFNWLEASTGGSILPQARNFPGFNRSRNVGSMSLSSTADDMAG-----
NAM-B2  253 DGLLTAEDAGLSAGPTSLSHLAAAARASPAPTKQFLAPSSSTPFNWLDASTVG-ILPQARNFPGFNRSRNVGNMSLSSTADMA------V
NAM-D1  265 DGLFTADDAGLSAGATSLSHLAPAARASPAPTKQFLAPSSSTPFNWLEASAAG-ILPQARNFPGFNRSRNVGNMSLSSTADMAG----AA
NAM-D2  253 DGLLTAEDAGLSAGATSLSHLAAAARASPAPTKQFLAPSSSTPFNWLDASTVG-ILPQARNFPGFNRSRNVGNMSLSSTADMA------V
NAM-H2  253 DGLLTAEDAGLSAGATSLSHLAAAARGSPAPTKQFLAPSSSTQFNWLDASTVG-ILPHARNFPGFNRSRNVGNMSLSSTADMAGAGTCAV
NAM-V1  261 DGLLTADNDGISAGATSLSHLAAAARASPAPTKQFLAPSSSTPFNWLDEPTAG-ILPQARNFHGFNRSRNVGNMSLSSTADMAG----AV

NAM-A1  348 G----NAVNAMSAFMNPLPVQDGTYHQHHVILGAPLAPEATTGGATSGFQHP-VQVSGVNWNP
NAM-B1  349 DN---GGGNAVNAMSTYLPVQDGTYHQQHVILGAPLVPEAAA--ATSGFQHP-VQISGVNWNP
NAM-H1  348 ---AVDVSDGGNAVNAMYLPVQDGTYHQHVILGAPLAPEAIAGAATSGFQHH-VQISGVNWNP
NAM-B2  336 DNGGGNAINTMPPFMNHLPMQDGTYHQQHVILGAPLAPEATAAATSAFQHP--VQISGVNWNP
NAM-D1  350 G----NAVNAMSAFMNPLPVQDGTYHQHHVILGAPLAPEATAGAATSGFQHHAVQISGVNWNP
NAM-D2  336 DNGGGNAINAMPPFMNHLPVQDGTYHQQHVILGAPLAPEATAAATSAF---------------
NAM-H2  342 DNGGGNAMNVMPPFMNHLPVQDGTYHQQHVILGAPLAPEATGAAASAFQHP--VQISGVNWNP
NAM-V1  346 DNGGGNAVNAMSAFMNPLPVQDGTYHHHHVILGAPVTPEATGGAATSGFQHP-VQISGVNWNP


   Fig. S1 Multiple alignments of deduced amino acid sequences of NAM proteins
